# Synergistic effect of clinically used antibiotics and peptide antibiotics against Gram-positive and Gram-negative bacteria

**DOI:** 10.3892/etm.2013.1231

**Published:** 2013-07-23

**Authors:** YULING ZHOU, YAN PENG

**Affiliations:** Department of Respiratory Medicine, Xinxiang Central Hospital of Henan Province, Xinxiang, Henan 453000, P.R. China

**Keywords:** Gram-positive aerobic bacteria, Gram-negative aerobic bacteria, synergy, peptide antibiotics

## Abstract

Ribosomally synthesized (natural) peptides demonstrate antimicrobial potency and may represent a novel therapeutic approach for the treatment of infections. The aim of the present study was to investigate the interaction between polycationic peptides and clinically used antimicrobial agents in the treatment of clinical isolates of Gram-positive and Gram-negative aerobic bacteria *in vitro,* using the microbroth dilution method. The combination studies demonstrated synergies between ranalexin and polymyxin E, doxycycline and clarithromycin. Similarly, magainin II was demonstrated to be synergistic with ceftriaxone, amoxicillin clavulanate, ceftazidime, meropenem, piperacillin and β-lactam antibiotics. Buforin II, cecropin P1 and indolicidin were not observed to be synergistic with the clinically used antibiotics, but demonstrated additive effects with them. Notably, no antagonistic effects were identified in all the combinations examined.

## Introduction

The discovery of numerous peptide antibiotics has resulted in a novel area of research into antimicrobial agents. In particular, ribosomally synthesized (natural) peptides, due to their antimicrobial potency, may represent a novel therapeutic approach for the treatment of infections (1–4). Antimicrobial peptides are produced in nature as a major component of the natural host defense molecules of a wide range of animals, plants and bacterial species (2,3,5–9). Studies have demonstrated that this group of peptides possesses a broad spectrum of antibacterial activity, the site of action of which is the cytoplasmic membrane (2,3,10–13). Various mechanisms have been suggested to explain the mode of action of these compounds on the membranes of bacteria. These mechanisms include the binding of monomers to the membrane and insertion into the membrane to form an ion-channel pore that spans the membrane; and the carpet model in which peptide molecules saturate the surface of the membrane prior to extensively disrupting the permeability barrier (1,2). The lethal event that occurs at the cytoplasmic membrane is not fully understood; however, it has been indicated that peptides cause channel formation in the cytoplasmic membrane, resulting in cell death (2,3,11,13,14). In addition, it has been shown that peptides allow maximal entry of several hydrophobic substrates into the cell and exert synergistic effects with lipophilic and amphiphilic agents, including rifampin, macrolides, fusidic acid and novobiocin (13,15).

The present study investigated the interaction between five polycationic peptides and several clinically used antibiotics in the treatment of clinical isolates of Gram-positive and Gram-negative bacteria *in vitro*.

## Materials and methods

### Bacteria

A total of 120 non-duplicate, clinical isolates were tested. These consisted of methicillin-susceptible *Staphylococcus aureus* (MS *S. aureus*; 30 strains), methicillin-resistant (MR) *S. aureus* (30 strains), *Pseudomonas aeruginosa* (30 strains) and *Escherichia coli* (30 strains). *S. aureus* ATCC 25923, *S. aureus* ATCC 38591 (oxacillin-resistant), *E. coli* ATCC 25922 and *P. aeruginosa* ATCC 27853 were used as control strains (The Institute of Microbiology, Chinese Academy of Science, Beijing, China).

### Antibacterial susceptibility testing

The minimal inhibitory concentrations (MICs) of all compounds were determined using a microbroth dilution method with Mueller-Hinton broth (Becton Dickinson Italia, Milan, Italy) and an initial inoculum of 5×10^5^ cfu/ml. The tests were conducted according to the procedures outlined by the National Committee for Clinical Laboratory Standards (16). Polystyrene 96-well plates (Becton Dickinson, Franklin Lakes, NJ, USA) were incubated for 18 h at 35°C in air. The MIC was considered to be the lowest drug concentration at which observable growth was inhibited.

### Interaction studies

In the interaction studies, the four control strains and two representative strains for each species of Gram-negative and Gram-positive bacteria were selected. The representative clinical isolates of the former (MS *S. aureus* 56–96, MR *S. aureus* 15–99, *E. coli* 31–97 and *P. aeruginosa* 8–97) showed the lowest MIC values for all peptides, and those of the latter (MS *S. aureus* 14–99, MR *S. aureus* 21–99, *E. coli* 7–98 and *P. aeruginosa* 17–99) showed the highest MIC values. The organisms were tested using the checkerboard titration method in 96-well polypropylene microtiter plates. The range of drug dilutions used was 0.125–64 *μ*g/ml for buforin II, cecropin P1, indolicidin, magainin II and ranalexin (Hangzhou Nuotai Co., Zhejiang, China). The peptides were solubilized in phosphate-buffered saline to provide 1 mg/ml stock solutions and 0.25–256 *μ*g/ml solutions of clinically used antibiotics were also prepared by dilution with phosphate-buffered saline. The clinically used antibiotics were vancomycin (Sigma, St. Louis, MO, USA), amoxicillin-clavulanate (SmithKline Beecham, Milan, Italy), clarithromycin (Abbott, Rome, Italy), meropenem (AstraZeneca, Rome, Italy), doxycycline, ceftazidime, polymyxin E and piperacillin (Wyeth-Lederle, Aprilia, Italy). Laboratory standard powders were diluted in accordance with the manufacturers’ recommendations. Drug solutions were prepared on the day of assay or stored in the dark at −80°C for short time periods. The amino acid sequences of the five peptides are shown in Table I. The fractionary inhibitory concentration (FIC) index for combinations of two antimicrobial agents was calculated according to the following equation: FIC index = FIC_A_+FIC_B_ = A/MIC_A_ + B/MIC_B_, where A and B are the MICs of drugs A and B, respectively, in the combination; MIC_A_ and MIC_B_ are the MICs of drugs A and B alone, respectively; and FIC_A_ and FIC_B_ are the FICs of drugs A and B, respectively. The FIC indices were interpreted as follows: ≤0.5, synergy; >0.5 to 1.0, addition; >1.0 to <4.0, indifference; and ≥4.0, antagonism (17).

## Results

The *in vitro* activities of buforin II, cecropin P1, indolicidin, magainin II and ranalexin against different bacteria are presented in Table II. The peptides demonstrated various ranges of inhibitory values in the different species of bacteria. Overall, Gram-negative strains were more susceptible to buforin II and cecropin P1, but less susceptible to ranalexin. Furthermore, *E. coli* strains were highly susceptible to the peptides, whereas the isolates of *P. aeruginosa* were scarcely susceptible. Buforin II and cecropin P1 both demonstrated MIC_50_ values of 0.50 *μ*g/ml for *E. coli* and 8 *μ*g/ml for *P. aeruginosa*. However, buforin II and ranalexin were the most active compounds against the staphylococcal isolates. They inhibited MS *S. aureus* strains at concentrations of 0.5–8 and 0.25–8 *μ*g/ml, respectively, and MR *S. aureus* strains at concentrations of 1–8 *μ*g/ml.

The combination studies indicated synergies between ranalexin and polymyxin E, doxycycline and clarithromycin, and the FIC indices aid in the quantification of the degree of synergy. The FIC indices demonstrated that the activity of ranalexin combined with polymyxin E, doxycycline or clarithromycin was four- to eight-fold greater compared with that of ranalexin alone (Tables III and IV). Furthermore, magainin II demonstrated synergistic effects with ceftriaxone, amoxicillin-clavulanate, ceftazidime, meropenem and piperacillin (Tables III and IV). The β-lactam antibiotics amoxicillin-clavulanate, ceftriaxone and meropenem decreased the MIC value of magainin II by a factor of eight (from 8 to 1 *μ*g/ml) for the oxacillin-resistant control strain of *S. aureus,* ATCC 38591. Moreover, the FIC indices showed that ranalexin and magainin II acted synergistically with clinically used antibiotics against Gram-positive and Gram-negative organisms (Tables III and IV). Additive effects, but no synergy, were demonstrated by combinations of buforin II, cecropin P1 and indolicidin with the clinically used antibiotics. Notably, antagonism was not identified in the combinations studied (data not shown).

## Discussion

Gram-negative and Gram-positive bacteria cause severe infectious diseases in mammals, and the emergence of their antimicrobial resistance is an increasing problem in human medicine. Throughout nature, polycationic peptides represent a conserved theme in antimicrobial defense. They may provide a new structural class of highly active antimicrobial agents, and potentially act as a resource for the development of novel anti-infective agents. Studies have indicated that polycationic peptides have variable antibacterial, antifungal and antiprotozoal activity *in vitro* (3,7,18–22). Therefore, these compounds may be very valuable as adjuvants for antimicrobial chemotherapy. Furthermore, studies have demonstrated that to exert their antimicrobial and anti-endotoxic activity, the peptides must initially bind to lipopolysaccharides (LPSs) of the outer membrane of Gram-negative bacteria (2,3,13). LPSs trigger the acute phase response to infection and the molecules involved in the recognition process have been extensively studied (9,23,24). However, comparatively little is known concerning the bioactive components of Gram-positive bacteria, and the mechanisms through which target cells are activated by these components. Peptidoglycan is a major component of the Gram-positive cell wall and does not contain LPS. However, it has been identified to trigger various defensive responses against bacterial infections (9,25).

The present study confirmed positive activity of each peptide against Gram-negative or Gram-positive bacteria. The combination studies demonstrated synergy between ranalexin and hydrophobic antibiotics, including polymyxin E, doxycycline and clarithromycin. In addition, magainin II was demonstrated to be synergistic with β-lactams. However, the mechanism of these positive interactions appears to be complex.

Ranalexin, a 20-residue peptide, demonstrates structural similarity to the polymyxins, a class of membrane-active antibiotics (8). The polymyxins are a group of cyclic polycationic peptides originally derived from *Bacillus polymyxa*. Similar to the polymyxins, ranalexin is an amphipathic compound with a cationic heptapeptide ring at its carboxyl terminus (the molecule contains two cysteine residues in positions 14 and 20, linked by a disulfide bridge) and a hydrophobic region at its amino terminus. Polymyxins and polymyxin-like peptides act synergistically with lipophilic and amphiphilic agents, including rifampin, macrolides, fusidic acid and novobiocin (10,12). Furthermore, it has been demonstrated that polymyxin-like peptides allow maximal entry of hydrophobic substrates into the cell (15).

The positive interaction between magainin II and β-lactam antibiotics may be due to an increased access of magainin II to the cytoplasmic membrane, following the breakdown of peptidoglycan by the β-lactam. However, other mechanisms may be involved in this interaction; the magainins may be membrane disruptive (6) and yield to the uncoupling of oxidative respiration (3,13). The peptide sequence of magainin II reveals that the molecule may exhibit large hydrophobic moments. When the peptide adopts an α-helical conformation in the solution, it is strongly amphiphilic, exhibiting a hydrophobic surface on one face and a hydrophilic surface on the other. It has been demonstrated that magainins are extremely surface active (6).

The reason for the lack of positive interactions between the other cationic peptides and the clinically used antibiotics tested in the current study is unclear. Cecropin P1 is a 31-residue peptide first isolated from the small intestine of pigs, with a strong basic N-terminal associated with a neutral C-terminal by a flexible glycine-proline link (20). Studies have identified several peptides, including cecropins and defensins, that resemble cationic detergents and lack synergy with hydrophobic antibiotics, but have direct antibacterial activity (12). Indolicidin, the smallest of the compounds tested, is a 13-amino acid antimicrobial peptide present in the cytoplasmic granules of bovine neutrophils (14). As a naturally occurring molecule, indolicidin has a unique composition consisting of 39% tryptophan and 23% proline, and is amidated at the C-terminus. Its particular composition and small dimensions make a comparison of its mechanism of action with that of other polycationic molecules difficult.

Studies have identified that buforin II may interact with biological membranes, in a similar manner to magainin; however, the mechanism of membrane interaction may be different. The length of its amphipathic region is ∼24 residues, which is approximately two-thirds that of magainin II (35 residues). The amphipathic region of buforin II is unable to span the whole biological membrane (the hydrophobic region is >30 residues); therefore, it is not possible to directly apply the ion channel model that was suggested for magainin II to this peptide (26).

In conclusion, a number of polycationic peptides have been demonstrated to bind to the LPSs of Gram-negative bacteria and to self-promote their uptake into bacteria (3). However, some of these molecules have markedly lower affinities for LPS binding, but are still effective permeabilizers, potentially through a related but distinguishable method (3).

The results regarding the activity of clarithromycin against Gram-negative organisms are particularly noteworthy. Macrolides may inhibit protein synthesis by binding to the transpeptidation site of the larger ribosomal subunit. However, large hydrophobic antibiotic molecules such as macrolides are mostly ineffective against Gram-negative bacteria, as they are unable to diffuse across the outer membrane (9,27,28). Notably, the present study identified that clarithromycin and ranalexin had synergistic activity against Gram-negative bacteria, including *P. aeruginosa*. There is increasing support for the activity of macrolides as antipseudomonal agents. Studies have suggested that macrolides may inhibit *P. aeruginosa* cell growth *in vitro*; although clarithromycin and erythromycin (at 2 mg/l) demonstrated no effect on cell growth at 24 h, they were bactericidal against *P. aeruginosa* when the incubation was continued for 48 h (24).

Multiple drug resistance is widespread among Gram-negative and Gram-positive bacteria. Therefore, there has been interest in the development of single drugs or drug combinations that have activity against multiple microorganisms. Novel drug combinations, prior to use in humans, must be tested *in vitro* and in experimental systems for enhanced activity and evidence of toxicity. Thus, to permit the full exploitation of polycationic peptides as novel antimicrobial agents, it is important to investigate their interaction with the most common clinically used antibiotics. Regardless of the speculated mode of peptide interactions, support for the clinical benefits is lacking. However, the data in the present study, concerning their intrinsic antibacterial activity and their synergistic interactions in several combinations, support the hypothesis that polycationic peptides may represent a novel and valuable adjuvant for antimicrobial chemotherapy.

## Figures and Tables

**Table I.** Primary structures of the five peptides.

Peptide	Amino acid sequence
Buforin II	Thr-Arg-Ser-Ser-Arg-Ala-Gly-Leu-Gln-Phe-Pro-Val-Gly-Arg-Val-His-Arg-Leu-Leu-Arg-Lys
Cecropin P1	Ser-Trp-Leu-Ser-Lys-Thr-Ala-Lys-Lys-Leu-Glu-Asn-Ser-Ala-Lys-Lys-Arg-Ile-Ser-Glu-Gly-Ile-Ala-Ile-Ala-Ile-Gln-Gly-Gly-Pro-Arg
Indolicidin	Ile-Leu-Pro-Trp-Lys-Trp-Pro-Trp-Trp-Pro-Trp-Arg-Arg
Magainin II	Gly-Ile-Gly-Lys-Phe-Leu-His-Ser-Ala-Lys-Lys-Phe-Gly-Ala-Phe-Val-Gly-Glu-Ilu-Met-Asn-Ser
Ranalexin	Phe-Leu-Gly-Gly-Leu-Ile-Lys-Ile-Val-Pro-Ala-Met-Ile-Cys-Ala-Val-Thr-Lys-Lys-Cys

**Table II.** *In vitro* susceptibilities to polycationic peptides.

Strains (no)	Agent	MIC (*μ*g/ml)		
		Range	50%^a^	90%^a^
*Escherichia coli* (30)	BF II	0.125–2	0.5	1
	C P1	0.125–2	0.5	2
	IND	0.5–8	2	4
	MG II	0.25–2	0.5	2
	RNL	1–32	4	16
*Pseudomonas aeruginosa* (10)	BF II	1–16	8	8
	C P1	2–32	8	16
	IND	4–64	16	64
	MG II	4–16	8	16
	RNL	8–64	16	32
MS *Staphylococcus aureus* (10)	BF II	0.5–8	2	8
	C P1	8–64	16	64
	IND	1–8	4	8
	MG II	8–64	16	64
	RNL	0.25–8	2	8
MR *Staphylococcus aureus* (10)	BF II	1–8	2	8
	C P1	16–64	32	>64
	IND	2–16	8	16
	MG II	8–64	32	64
	RNL	1–8	4	8

a MICs at which 50 and 90% of the strains are inhibited; MS, methicillin-susceptible strains; MR, methicillin-resistant strains; MIC, minimun inhibitory concentration; BF II, buforin II; C P1, ceropin P1; IND, indolicidin; MG II, magainin II; RNL, ranalexin.

**Table III.** FIC indices from interaction studies between polycationic peptides and clinically used antibiotics.

Agent	*S. aureus* ATCC 25923		MS *S. aureus* 56–96		MS *S. aureus* 14–99		*S. aureus* ATCC 38591		MS *S. aureus* 15–99		MS *S. aureus* 21–99	
					
	MG II	RNL	MG II	RNL	MG II	RNL	MG II	RNL	MG II	RNL	MG II	RNL
AMC	0.187	1.000	0.250	1.250	0.187	1.000	0.312	1.500	0.312	1.500	0.250	1.250
CRO	0.312	0.750	0.250	1.500	0.250	1.000	0.250	1.000	0.250	1.250	0.250	1.500
MEM	0.187	1.000	0.375	1.250	0.375	1.250	0.250	1.500	0.187	1.500	0.187	1.500
D	1.000	0.250	0.750	0.312	1.000	0.312	1.000	0.187	0.750	0.250	0.750	0.187
CLR	0.750	0.250	1.000	0.312	0.750	0.250	1.000	0.250	0.750	0.187	0.750	0.250

FIC, fractionary inhibitory concentration; *S. aureus*, S*taphylococcus aureus;* MS, methicillin-susceptible; MG II, magainin II; RNL, ranalexin; AMC, amoxicillin-clavulanate; CRO, ceftriaxone; MEM, meropenem; D, doxycyline and CLR, clarithromycin.

**Table IV.** FIC indices from interaction studies between polycationic peptides and clinically used antibiotics (continued).

Agent	*P. aeruginosa* ATCC 27853		*P. aeruginosa* 8–97		*P. aeruginosa* 17–99		*E. coli* ATCC 25922		*E. coli* 31–97		*E. coli* 7–98	
					
	MG II	RNL	MG II	RNL	MG II	RNL	MG II	RNL	MG II	RNL	MG II	RNL
AMC	0.187	1.000	0.187	1.000	0.187	0.750	0.187	1.500	0.312	1.250	0.187	1.000
PIP	0.250	0.750	0.187	1.000	0.250	1.000	0.312	0.750	0.312	1.250	0.250	1.000
CAZ	0.250	1.250	0.375	1.500	0.250	1.000	0.187	1.250	0.187	1.250	0.312	1.250
CRO	0.250	1.000	0.312	0.750	0.250	1.250	0.250	1.000	0.312	1.250	0.250	1.250
MEM	0.187	0.750	0.250	1.000	0.312	0.750	0.312	0.750	0.375	1.000	0.375	1.500
CLR	1.000	0.250	1.500	0.375	1.500	0.250	1.500	0.375	1.500	0.312	1.500	0.312
D	1.500	0.250	1.500	0.312	2.000	0.375	1.500	0.312	1.250	0.375	2.000	0.312
POL-E	1.250	0.250	1.250	0.250	1.000	0.250	1.500	0.187	1.500	0.250	1.250	0.312

FIC, fractionary inhibitory concentration; *P. aeruginosa, Pseudomonas aeruginosa; E. coli, Escherichia coli;* MG II, magainin II; RNL, ranalexin; AMC, amoxicillin-clavulanate; PIP, piperacillin; CAZ, ceftazidime; CRO, ceftriaxone; MEM, meropenem; CLR, clarithromycin; D, doxycycline; POL-E, polymyxin E.
